# Quantifying the effect of dobutamine stress on myocardial Pi and pH in healthy volunteers: A ^31^P MRS study at 7T

**DOI:** 10.1002/mrm.28494

**Published:** 2020-09-14

**Authors:** Andrew Apps, Ladislav Valkovič, Mark Peterzan, Justin Y. C. Lau, Moritz Hundertmark, William Clarke, Elizabeth M. Tunnicliffe, Jane Ellis, Damian J. Tyler, Stefan Neubauer, Oliver J. Rider, Christopher T. Rodgers, Albrecht Ingo Schmid

**Affiliations:** ^1^ Oxford Centre for Clinical Magnetic Resonance Research (OCMR) Division of Cardiovascular Medicine, Radcliffe Department of Medicine University of Oxford Oxford United Kingdom; ^2^ Department of Imaging Methods Institute of Measurement Science Slovak Academy of Sciences Bratislava Slovakia; ^3^ Department of Physiology, Anatomy and Genetics University of Oxford Oxford United Kingdom; ^4^ Wolfson Brain Imaging Centre University of Cambridge Cambridge United Kingdom; ^5^ High Field MR Center, Center for Medical Physics and Biomedical Engineering Medical University of Vienna Vienna Austria

**Keywords:** 7T, cardiac energetics, myocardial pH, ^31^P MRS, STEAM

## Abstract

**Purpose:**

Phosphorus spectroscopy (^31^P‐MRS) is a proven method to probe cardiac energetics. Studies typically report the phosphocreatine (PCr) to adenosine triphosphate (ATP) ratio. We focus on another ^31^P signal: inorganic phosphate (Pi), whose chemical shift allows computation of myocardial pH, with Pi/PCr providing additional insight into cardiac energetics. Pi is often obscured by signals from blood 2,3‐diphosphoglycerate (2,3‐DPG). We introduce a method to quantify Pi in 14 min without hindrance from 2,3‐DPG.

**Methods:**

Using a ^31^P stimulated echo acquisition mode (STEAM) sequence at 7 Tesla that inherently suppresses signal from 2,3‐DPG, the Pi peak was cleanly resolved. Resting state UTE‐chemical shift imaging (PCr/ATP) and STEAM ^31^P‐MRS (Pi/PCr, pH) were undertaken in 23 healthy controls; pH and Pi/PCr were subsequently recorded during dobutamine infusion.

**Results:**

We achieved a clean Pi signal both at rest and stress with good 2,3‐DPG suppression. Repeatability coefficient (8 subjects) for Pi/PCr was 0.036 and 0.12 for pH. We report myocardial Pi/PCr and pH at rest and during catecholamine stress in healthy controls. Pi/PCr was maintained during stress (0.098 ± 0.031 [rest] vs. 0.098 ± 0.031 [stress] *P* = .95); similarly, pH did not change (7.09 ± 0.07 [rest] vs. 7.08 ± 0.11 [stress] *P* = .81). Feasibility for patient studies was subsequently successfully demonstrated in a patient with cardiomyopathy.

**Conclusion:**

We introduced a method that can resolve Pi using 7 Tesla STEAM ^31^P‐MRS. We demonstrate the stability of Pi/PCr and myocardial pH in volunteers at rest and during catecholamine stress. This protocol is feasible in patients and potentially of use for studying pathological myocardial energetics.

## 1 INTRODUCTION

The beating heart is powered by chemical potential energy (ΔG_ATP_) released during the hydrolysis of adenosine triphosphate (ATP) into the products adenosine diphosphate (ADP) and inorganic phosphate (Pi). Instantaneous myocardial ATP content is low and only sufficient to sustain a few beats.^1^ Maintenance of [ATP] local to the myofibril is achieved by rapid phosphotransfer from a reservoir of phosphocreatine (PCr) to ADP, catalyzed by the enzyme creatine kinase (CK).^2^ The heart’s energetic state can be summarized via the relative concentrations of these high‐energy phosphate metabolites, which can be measured in vivo using phosphorus MR spectroscopy (^31^P‐MRS).

Much of the existing ^31^P‐MRS literature has focused on reporting the PCr/ATP ratio as a measure of myocardial energetics. Resting PCr/ATP is reduced not only in heart failure^3^, ^4^ but also in systemic conditions at risk of developing a cardiomyopathy phenotype, such as obesity^5^, ^6^ and diabetes.^7^ Abnormal energetics confer a poor prognosis,^3^ and as such represent an attractive therapeutic target with a need to develop further tools to assess it. However, focusing solely on PCr/ATP can mask reductions in both PCr *and* ATP concentration in progressive heart failure.^8^, ^9^, ^10^ Pathological energy metabolism is characterized by an abnormal response to stress. In the healthy heart, PCr/ATP is maintained within the cardiac cycle^11^ and during incremental stress,^5^, ^7^, ^12^, ^13^, ^14^ whereas pathological energy metabolism, evidenced by a reduced ratio, is often exacerbated or uncovered during such stress.^5^, ^7^, ^13^ Novel techniques to study the high‐energy phosphate pool should thus be acceptable for use during pharmacological stress.

In myocardium, free ADP concentration is too low (20‐60 µM) to observe directly by ^31^P‐MRS in vivo,^15^ Pi, however, can be (~1 mM) and is an attractive candidate for assessing metabolism in vivo for a variety of reasons.^16^, ^17^ Firstly, Pi plays an important signalling role in the control of oxidative phosphorylation, ensuring that ATP regeneration responds to an increase in demand. Pi stimulates oxygen consumption in isolated mitochondria,^18^ and various in‐silico models directly implicate the small increase in cellular Pi consequent to increased ATP hydrolysis at high workloads as a crucial mechanism to upregulate oxidative phosphorylation and maintain ΔG_ATP_.^19^, ^20^, ^21^ Secondly, a rise in [Pi] has been modeled to follow a reduction in the forward CK rate constant,^22^ which is shown to be reduced by up to 50% in heart failure.^23^, ^24^ Pi may thus represent a proxy through which CK flux can be investigated. Indeed, Pi is predicted to increase over 3× during exercise in hypertrophic cardiomyopathy by a biophysical model of energy metabolism used to model the energetic response to exercise in the condition. These increases were further exacerbated by creatine loss.^25^ Additionally, Pi/PCr may offer a more sensitive marker of early pathological energy metabolism than PCr/ATP. Pi is implicated in the initial increase in glucose oxidation seen in early compensated heart failure^26^; it stimulates both glycolysis and glycogenolysis^27^ and is shown to be elevated in hypertrophy.^28^, ^29^ For example, Pi/PCr was shown to increase during intracardiac pacing in dogs with left ventricular hypertrophy but not in controls, whereas PCr/ATP remained stable in both groups.^30^ Finally, intracellular pH can be calculated from the frequency difference between Pi and other metabolites. A fall in pH is a clinically relevant parameter heralding contractile dysfunction and dysrhythmia.^31^

**FIGURE 1:**
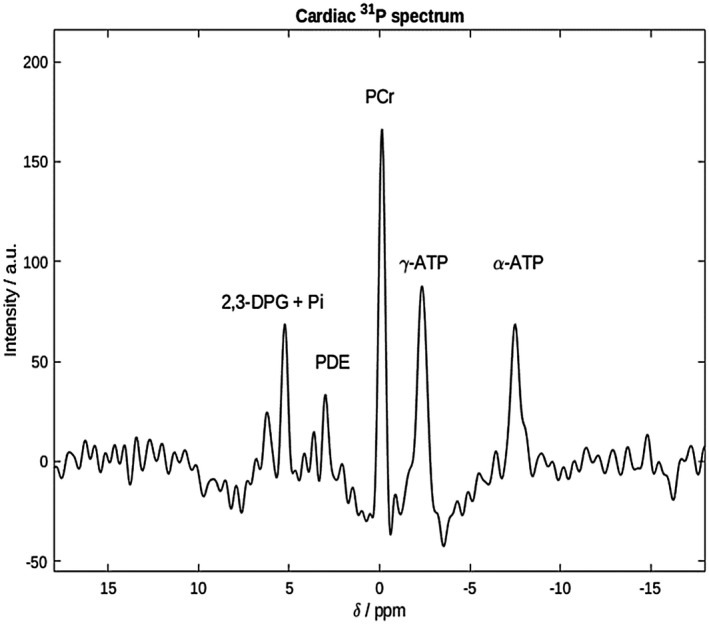
Cardiac 7T ^31^P spectrum from the interventricular septum of a healthy participant. PCr, γ‐ and α‐ATP, PDE, and 2,3‐DPG are clearly seen. Pi is not visible, being obscured by the 2,3‐DPG peak at around 5 ppm. The excitation bandwidth was too small to excite β‐ATP 2,3‐DPG, 2,3‐diphosphoglycerate; ^31^P, phosphorus; ATP, adenosine triphosphate; PCr, phosphocreatine; PDE, phosphodiesters; Pi, inorganic phosphate

Data on human cardiac Pi are scarce because overlap from 2,3‐diphosphoglycerate (2,3‐DPG) has made it challenging to quantify reliably (Figure 1). The myocardium forms a comparatively thin wall around the blood‐filled ventricle, and resolving blood from heart muscle tissue by localization techniques is not possible given the voxel size required for adequate ^31^P‐MRS SNR. Various ^31^P‐MRS approaches have been suggested to measure Pi in the presence of contaminating blood pool 2,3‐DPG. We have recently reported Pi using a long‐T_R_ chemical shift imaging (CSI) protocol.^32^ The defining feature was the favorable balance of myocardial Pi to blood 2,3‐DPG signal intensity in the case of long repetition times with adiabatic excitation. 2,3‐DPG peaks, however, were still present, to some extents still obscuring Pi, and sampling was very long (45 min), rendering the protocol unsuitable for use during vasoactive stress studies. Other approaches have been tried in the past at lower field strengths, including hydrogen‐1 (^1^H)‐decoupled ^31^P‐MRS to reduce Pi and 2,3‐DPG linewidths in the hope of achieving separated peaks^33^, ^34^; Pi was reported only for about half the healthy subjects. This is infeasible at 7T due to regulatory limits on specific absorption rate and the restriction on peak $B_{1}^{+}$ for ^1^H.

We therefore aimed at developing a measurement technique for Pi that provides both an adequate SNR within a scan time that allows stress measurements in both controls and patients and good blood signal suppression to allow reliable quantification of Pi amplitude and frequency (pH). Stimulated echo acquisition mode (STEAM) has been previously shown to suppress signal from moving spins in blood in ^1^H‐MRI^35^, ^36^ and is also a commonly used and available MRS technique. In contrast to CSI,^37^ which generates gradient echo‐like bright blood contrast, STEAM generates dark‐blood contrast. We investigated a 14‐min STEAM protocol to demonstrate cardiac Pi without obstruction at rest but also during catecholamine stress, that is, at a new steady‐state. Given the potential impact of the mixing time (T_M_) on susceptibility of various factors such as SNR, heart rate, myocardial deformation, and SNR to impact measurements during T_M_, we first verified the repeatability of Pi/PCr and pH measurements in 9 control participants. We then tested the hypothesis that Pi/PCr and pH may change in response to increasing cardiac work in a healthy cohort of 23 healthy controls, and finally demonstrated feasibility in a patient with cardiomyopathy in preparation for future clinical studies.

## 2 METHODS

### 2.1 Ethics and study cohort

Healthy volunteers for intra‐exam repeatability studies were recruited according to our institutions ethics regulations prior to undertaking dobutamine stress studies. Research studies involving dobutamine stress at 7T were approved by the National Research Ethics Committee service (3/SC/0376), and written informed consent was obtained from each participant. Nine participants (2 female, age 37 ± 9 years) were recruited for repeatability experiments (of those, 1 was scanned on different days); 23 participants (8 female, age 41 ± 1 years, body mass index 24 ± 2 kg/m^2^) were subsequently recruited for the stress studies. Subjects were healthy; they were excluded if they were taking prescription medication; had a contraindication to 7T MR imaging (defined by institutional strandard operating procedures); or had a medical history of cardiovascular disease, peripheral vascular disease, diabetes mellitus, hypertension, or undiagnosed chest pain. All participants had a normal clinical cardiovascular examination and normal resting global cardiac function on cine ^1^H MR imaging. To demonstrate the feasibility for future patient studies, we recruited 1 patient with idiopathic dilated cardiomyopathy ([DCM] 55‐year old male, body mass index 26 kg/m^2^, left ventricular ejection fraction 25%, with no evidence of coronary artery disease on CT coronary angiography).

### 2.2 Pulse sequence design

**FIGURE 2:**
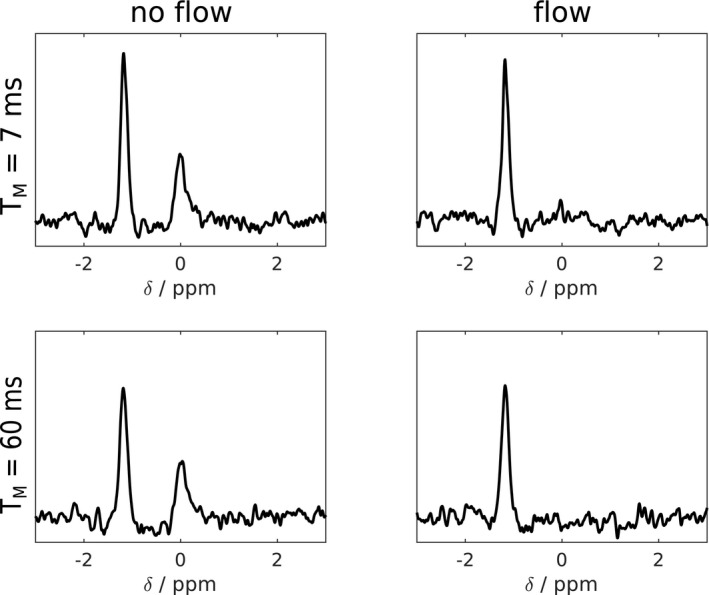
STEAM‐^31^P‐MR spectra are from a phantom containing 2 compartments with Pi of different pH. The outer compartment is stationary, whereas the inner compartment is connected to a pump outside of the scanner. When turning the pump on, the signal from the outer compartment is largely unaffected, whereas the inner compartment shows some residual peak with T_M_ of 7 ms while it is below noise level with T_M_ of 60 ms. This may not represent the optimal choice of T_M_; however, in agreement with previous data,^36^ we found that it was reasonable and worked well. STEAM, stimulated echo acquisition mode; T_M_, mixing time

**FIGURE 3:**
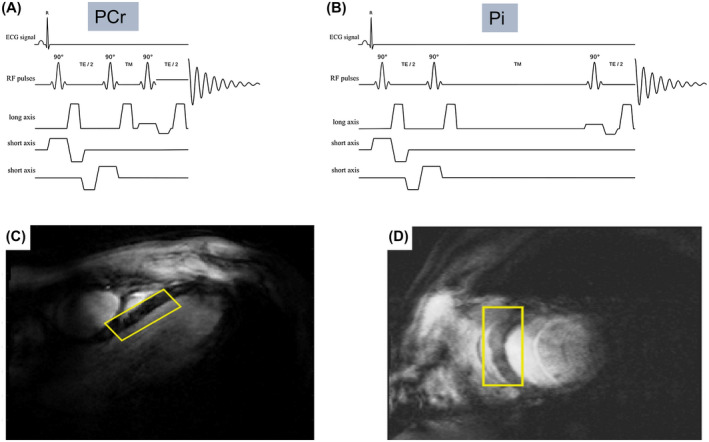
STEAM‐^31^P‐MRS sequence for interleaved RF excitation centered at PCr (A: RF excitation centred at 0 ppm relative to PCr, T_M_ 7 ms) and Pi (B: RF excitation centered at 4.8 ppm relative to PCr, T_M_ 60 ms). Typical STEAM‐^31^P‐MRS voxel placement ([C] horizontal long axis view, [D] short axis view); this was chosen to optimise septal coverage while minimizing the potential for skeletal muscle contamination

This work employed a STEAM sequence for ^31^P‐MRS at 7T. Unlike the gradient echo/FLASH‐like contrast of CSI sequences often used for cardiac ^31^P‐MRS, STEAM ^1^H cardiac imaging achieves dark blood contrast due to incoherent motion of the blood during T_M_, which leads to dephasing of the blood signal.^35^ Meanwhile, the myocardium is relatively immobile so that myocardial ^31^P signals are preserved if the T_M_ is chosen appropriately.^36^ The RF coil used had good receive sensitivity with 16 receive channels but a rather low transmit field (peak $B_{1}^{+}$ was about 10 µT at the depth of the heart), meaning that long (4.5 ms truncated sinc) pulses were required to obtain the 90° pulses required by STEAM. Such pulses have small bandwidth and, when compounded by large voxel dimensions, result in significant chemical shift displacement. We thus split the experiment into 2 excitations, centering the RF pulses on the PCr peak in the first scan and on Pi in the second scan. These two were interleaved during T_R_. This has an additional advantage that T_E_ and in particular T_M_ can be set to different values for Pi and PCr. Based on STEAM imaging data,^36^ a T_M_ of 60 ms was chosen for Pi, representing a good compromise between blood suppression and sensitivity to diffusion and cardiac motion that would cause dephasing and signal loss. A flow phantom comprised of both stationary and moving (3 mL/s) compartments, each containing Pi at different pH, was used to confirm that a T_M_ of 60 ms was a suitable choice prior to beginning the study. The signal from the flowing compartment could be adequately suppressed using the chosen parameter of T_M_ 60 ms, with little impact on the signal from the static compartment. In contrast, tests with a shorter T_M_ of 7 ms showed incomplete suppression of signals from the flowing compartment (~10% residual from the original signal), as shown in Figure 2. Recording of the stimulated echo for each metabolite was in early systole just following the isovolumetric contraction period^38^; the point of data collection within the cardiac cycle was thus fairly constant despite the heart rate increasing during stress. Scalar coupling during the prolonged T_M_ further helps suppress signals from 2,3‐DPG. We used the shortest possible echo time (T_E_ = 13 ms) to minimize effects of T_2_ relaxation on the Pi signal. T_1_ relaxation is practically irrelevant given the long relaxation times. PCr was acquired with short T_M_ (7 ms). The sequence is summarized in Figure 3.

### 2.3 ^31^P‐MRS protocols

All scanning was performed on a Magnetom whole‐body 7T MRI scanner (Siemens, Erlangen, Germany). Using a 10 cm ^1^H transmit/receive loop (Rapid Biomedical, Rimpar, Germany), localizers were acquired with 3‐lead ECG gating in the main cardiac orientations,^37^ and subject‐specific B_0_‐shimming was performed ensuring coverage of the whole heart.^39^ Localizers were acquired at the end of expiration, making contamination of the voxel of interest by subdiaphragmatic structures during our free‐breathing MRS protocols highly unlikely. With the participant asked to remain as still as possible, the table was moved out, and the ^1^H coil was then removed and swapped for a 16‐element array ^31^P coil (Rapid Biomedical, Rimpar, Germany). This comprised a rigid 27 × 28 cm^2^ transmit ^31^P element, combined with a flexible set of 16 receive elements (size 8 × 5.5 cm^2^, arranged in a 4 × 4 grid). ^31^P‐FLASH localisers were acquired from 5 fiducial markers mounted on the coil to determine the coil position, and a set of nonlocalized inversion recovery‐free induction decay scans (inversion recovery‐FIDs) was acquired for each participant. Using these, the coil $B_{1}^{+}$ map was calculated using the Biot‐Savart Law to estimate the required power to provide a 90° flip angle in the STEAM voxel placed over the cardiac septum. The unavoidable $B_{1}^{+}$ inhomogeneity of a surface transmitter leaves residual 20% to 30% variation in flip angle across the STEAM voxel. This approach for MRS calibration at 7T was previously described.^40^

First, PCr/γ‐ATP ratio was measured using a UTE‐CSI pulse sequence with T_R_ of 2.2 s, run at rest with free breathing, acquisition weighting (matrix size 8 × 16 × 6), and zero‐filled to 8 × 16 × 8 for reconstruction. The nominal voxel size was 25 × 15 × 33 mm^3^. Excitation was with a shaped pulse centred at +300 Hz relative to PCr.^39^ The CSI orientation was cardiac short axis in‐plane rotated so the phase‐encoding direction with the highest resolution was in the anteroposterior direction to minimize skeletal muscle contamination. Next, ECG‐triggered, free‐breathing STEAM‐^31^P‐MRS spectra were acquired with shot‐to‐shot interleaving of acquisitions centered: 1) on PCr (0 ppm, T_M_ = 7 ms) (Figure 3A, PCr interleaf), and 2) on Pi (4.8 ppm, T_M_ = 60 ms) (Figure 3B, Pi interleaf). A T_R_ of 3 s was used, with the first pulse of the next acquisition delivered on the subsequent R wave. This meant the effective repetition time for each metabolite was 2 × (T_R_ plus an intertrigger delay time) ≈ 7 s. 128 averages were acquired, with scans typically lasting 14 min. The long effective repetiton time ensured 80% to 90% recovery of magnetization between consecutive excitations, improving the detectability of static spins with long T_1_, such as cardiac Pi.^32^ Our STEAM‐^31^P‐MRS voxel placement (Figure 3C,D) ensured maximal septal coverage and minimal skeletal muscle contamination. Voxel size for STEAM was adjusted slightly between subjects (58 ± 12 mL).

### 2.4 Dobutamine infusion protocol including ^31^P‐STEAM MRS

Immediately following the resting state STEAM‐^31^P‐MRS acquisition, an intravenous dobutamine infusion (GRASEBY 3500 anaesthesia pump, Graseby Medical, Ashford, UK) was started at a dose of 5 µg/kg/min, increasing at regular intervals up to a maximum dose of 40 µg/kg/min to achieve a target heart rate of 65% of the age‐predicted maximal heart rate. During the infusion, participants had continuous electrocardiogram and heart rate monitoring by pulse oximetry, and noninvasive blood pressure monitoring (Vicorder, SMT Medical, Würzburg, Germany) every min. The mean rate pressure product (RPP = systolic blood pressure × heart rate) was recorded at rest and during stress. Heart rate was maintained at target for the duration of the stress, and the STEAM‐^31^P‐MRS measurement described above was repeated. The DCM patient also consented to a recovery STEAM‐^31^P‐MRS measurement when their heart rate had normalized after the infusion. All stress scans were overseen by 2 experienced operators (A.A., a clinician with 6 years of cardiology practice and 2 years of MR imaging; and either L.V., A.I.S., or J.E. with 8, 15, and 3 years of 7T ^31^P MRS experience, respectively).

### 2.5 Data analysis

**FIGURE 4:**
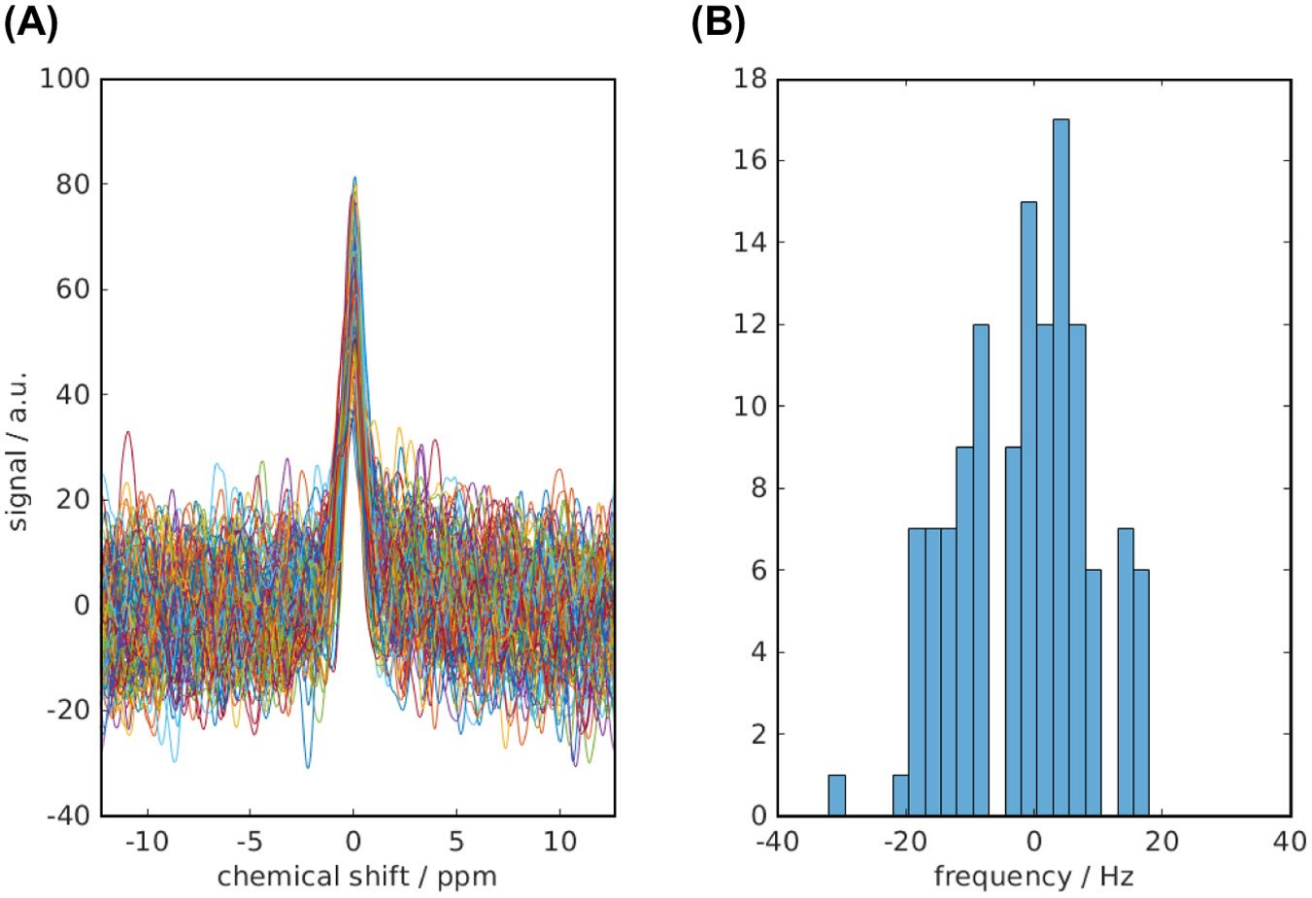
With separate excitations for PCr and Pi, frequency fluctuations had the potential to affect pH estimation. In 1 data set (128 averages), the individual un‐averaged FIDs were extracted from the raw data and are plotted after apodization with the fitted line width (A); fluctuations are seen to average toward 0 mean frequency offset. The frequency distribution also lies within the fitted line width (65 Hz) (B)

Coil combination was done using a whitened singular value decomposition on the PCr spectra.^41^ Fitting was performed using the open‐source MatLab‐based “OXSA” toolbox (MathWorks, Natick, MA).^42^, ^43^ UTE‐CSI spectra were fitted as previously described^40^ using 12 Lorentzian peaks for ATP, PCr, and 2,3‐DPG, with literature values for scalar couplings and initial frequency estimates. Peak amplitudes were corrected for partial saturation and blood contamination.^40^ We report the PCr/γ‐ATP ratio because α‐ATP overlaps with the reduced form of nicotinamide adenine dinucleotide, and β‐ATP was incompletely excited. To fit STEAM‐^31^P‐MRS spectra, first the PCr‐interleaf spectrum was fitted; then the Pi‐interleaf spectrum was fitted using 4 Lorentzian peaks at PCr, Pi, and 2 phosphodiester frequencies, signals typically found in these spectra. The algorithm fitting the Pi frequency was constrained to search between +4.2 and +5.8 ppm relative to PCr (frequency taken from the PCr interleaf); Pi and PCr linewidths were set to be equal to improve the stability of fitting. The PCr phases were used as starting points for the Pi parameter fitting, and both peaks were corrected for partial saturation using literature values.^40^, ^44^ Cramér‐Rao lower bounds^45^ were calculated for the Pi amplitudes, and pH was calculated from the Pi chemical shift (taken from the Pi interleaf) relative to that of PCr (taken from the PCr interleaf) using a modified Henderson–Hasselbalch relationship: $\text{pH}\left(\delta \right)=6.77+\log_{10}\frac{\delta ‐3.23}{5.70‐\delta }$.^46^ The chemical shift measurement required for pH estimation was taken from 2 separate excitations; however, systematic frequency shifts were extremely unlikely to happen with the same periodicity given the length of scan. Indeed, reassuringly over the 128 averages comprising each scan, frequency fluctuations of the PCr acquisition were found to effectively average toward 0 mean frequency offset (Figure 4A), with the distribution of frequency offsets lying within the fitted line width (65 Hz) (Figure 4B). To test the repeatability of cardiac Pi/PCr quantification and pH calculation, Bland‐Altman analysis of agreement was used to compare the 2 measurements, and coefficients of repeatability were estimated, as described by Bartlett et al, defined as $1.96\times \sqrt{2}\times within\;subject\;SD$.^47^

We sought to undertake a paired analysis of the effect of stress on Pi/PCr. Myocardial [Pi] is extremely low; thus, poor SNR at rest or any significant loss of SNR, as is sometimes encountered during dobutamine stress, may impact our ability to identify its resonance. Data were included for analysis if the Pi peak was clearly resolved (defined as SNR >2.5 *and* visually obvious). Details of evaluating data quality and inclusion from all participants in the study are given in the Supporting Information Table S1. Statistical analysis was performed in GraphPad Prism v6.01 (GraphPad, La Jolla, CA). Results are given as mean ± SD. Comparisons between rest and stress values for pH and Pi/PCr were undertaken using paired Student *t* tests.

## 3 RESULTS

### 3.1 Repeatability

**FIGURE 5:**
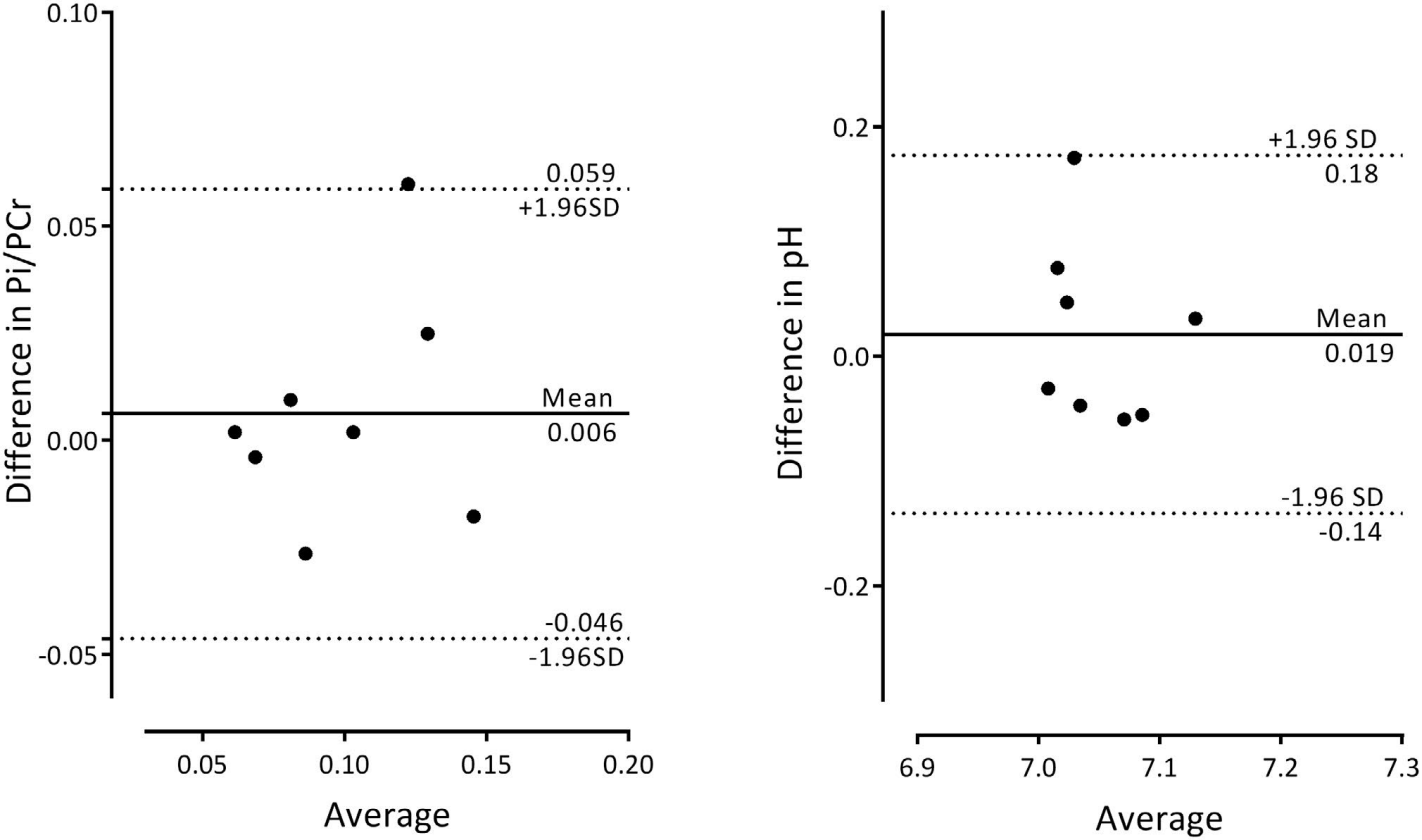
Bland Altman plots of intra‐exam repeated Pi/PCr and pH measurements. Solid lines demonstrate the bias (scan 1–scan 2, ideally 0). Dashed lines represent the limits of agreement, that is, representing ±1.96 SD. The coefficient of repeatability for Pi/PCr was 0.036 and was for pH 0.12

**FIGURE 6:**
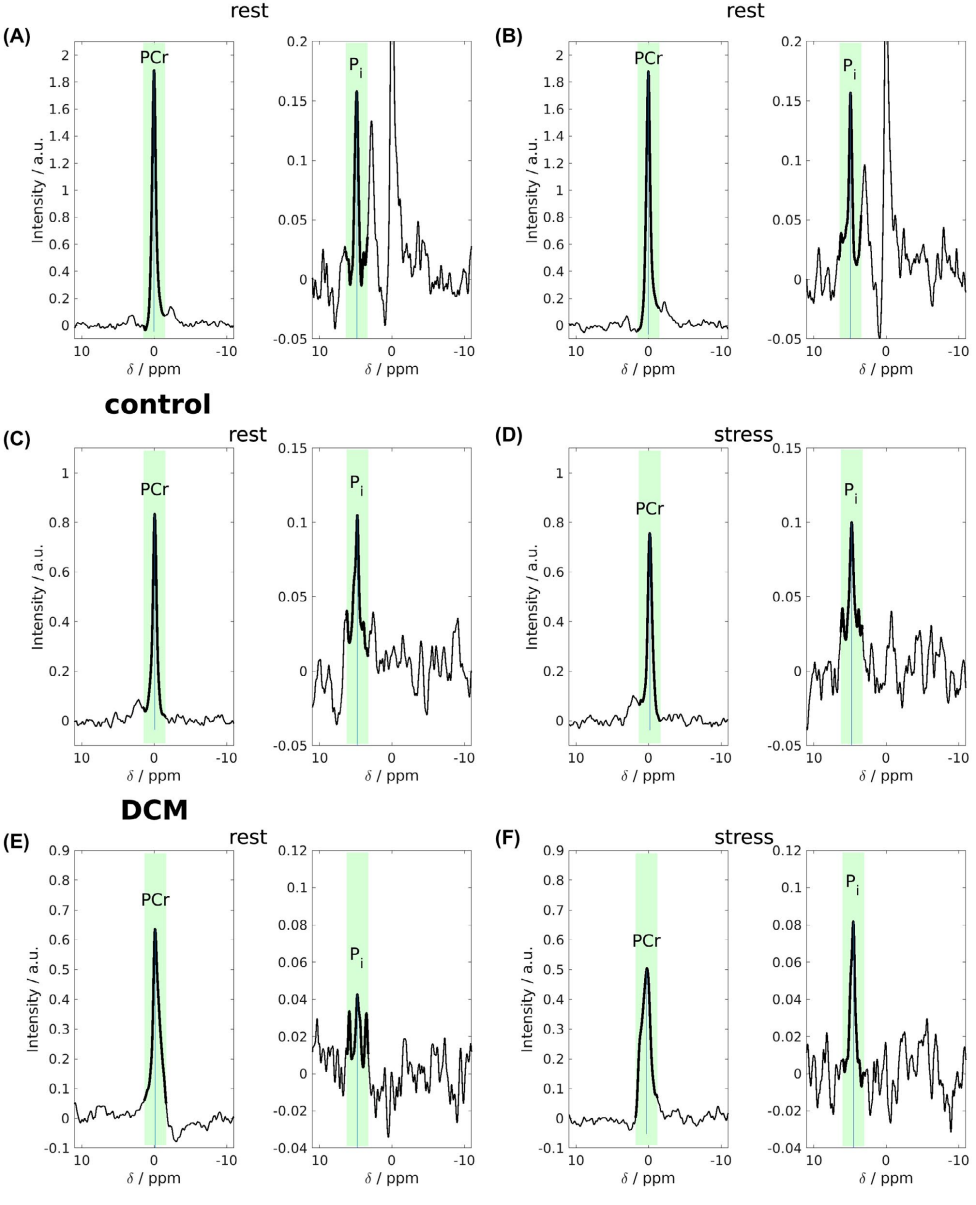
STEAM‐^31^P‐MRS spectra from the 2 interleaved acquisitions from different experiments. Repeatability scan data are shown (A,B) for a healthy volunteer. Rest (C,E) and dobutamine stress (D,F) data of another healthy volunteer (C,D) and the patient with DCM (E,F) are shown. In each interleave, the spectral region to be trusted is that immediately surrounding the metabolite excited marked by bolder lines and green background. Other parts of the spectrum arise from different spatial locations due to strong chemical shift displacement effects and cannot be taken as cardiac. Pi can clearly be resolved with little or no contaminating 2,3‐DPG. pH calculations are undertaken from the chemical shift of the Pi peak (taken from the Pi interleaf) relative to that of the PCr peak (taken from the PCr interleaf). DCM, dilated cardiomyopathy

Prior to investigating the effect of dobutamine on cardiac Pi/PCr and pH, to confirm the precision of measurements we performed intra‐exam repeatability studies at rest. Pi was clearly identified (by the above criteria) in 17 of 18 scans undertaken for repeatability. A representative fit of the Pi peak demonstrating the residual signal can be found in Supporting Information Figure S1. No significant differences were seen between scans 1 and 2 for Pi/PCr (0.103 ± 0.044 vs. 0.097 ± 0.039 *P* = .53 [*n* = 8]]) or pH (7.06 ± 0.05 vs 7.04 ± 0.06 *P* = .52 [*n* = 8]). The mean absolute bias between scans was +0.006 (Pi/PCr) and +0.02 (pH), with 95% limits of agreement of ±0.052 (Pi/PCr) and ±0.16 (pH). The coefficient of repeatability for Pi/PCr was 0.036, and that for pH 0.12. Results are demonstrated in a Bland‐Altman plot (Figure 5), with a typical pair of spectra shown in Figure 6A,B.

### 3.2 Resting myocardial energetics and pH in healthy controls

Mean blood‐ and saturation‐corrected PCr/γ‐ATP was recorded successfully in 22 of 23 subjects of this group and was 2.11 ± 0.45. Mean STEAM‐^31^P‐MRS SNR for PCr was 78 ± 42. Pi had a lower signal due to its low concentration; we were able to resolve Pi successfully in 20 of 23 subjects at rest by visual inspection and using a cutoff of SNR > 2.5 (6.7 ± 3.9) Average acquisition duration was 15.1 ± 0.9 min. 2,3‐DPG was not visible in any of the ^31^P STEAM spectra above the noise floor. Considering the 20 spectra with unambiguous peak detection, mean resting Pi/PCr 0.098 ± 0.030 and myocardial pH 7.07 ± 0.08.

### 3.3 Stress in healthy controls

**FIGURE 7:**
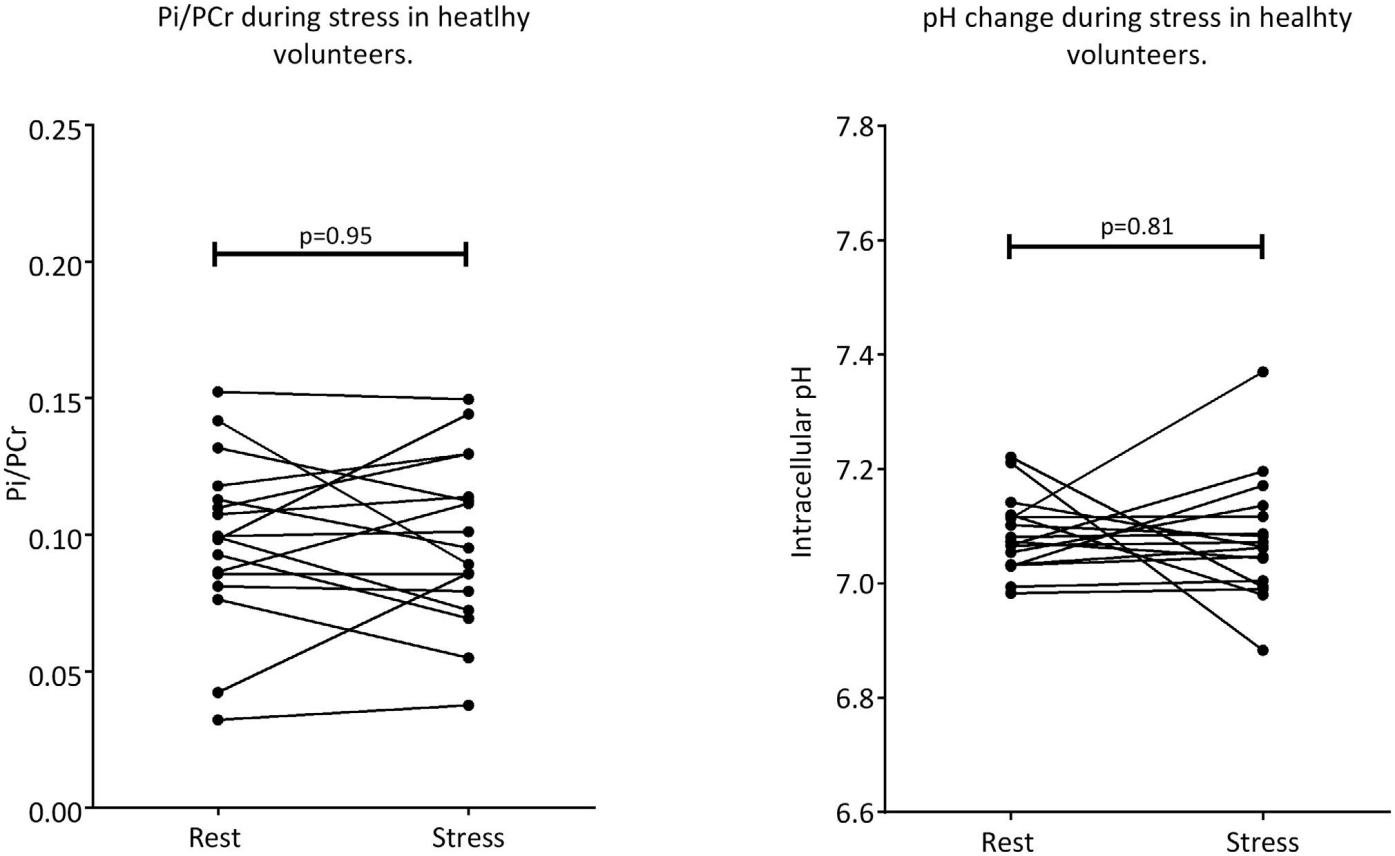
Myocardial Pi/PCr and pH were maintained in the physiologically normal range during moderate catecholamine stress in this healthy cohort

Twenty‐two of 23 healthy controls underwent successful STEAM‐^31^P‐MRS at stress (1 scan was aborted due to a failure of the blood pressure monitoring system). All dobutamine infusions were well tolerated. Mean STEAM‐^31^P‐MRS stress scan length was 13.6 ± 1.1 min. A typical pair of spectra from a healthy volunteer is shown in Figure 6C,D. Using the above‐described criteria for the resting state spectra, Pi was adequately identified in 18 of the 22 stress scans (stress Pi/PCr 0.096 ± 0.031, pH 7.08 ± 0.10). In 5 participants, Pi detection was not robust either at rest,^1^ stress,^2^ or both^2^ to justify inclusion in a paired analysis. This was undertaken on 17 datasets. More details can be found summarized in Supporting Information Table S1 and Supporting Information Figure S2. Pi/PCr was not significantly different during stress (0.098 ± 0.031 [rest] vs. 0.098 ± 0.031 [stress], *P* = .95). Similarly pH did not change (7.09 ± 0.07 [rest] vs. 7.08 ± 0.11 [stress], *P* = .81) (Figure 7). The median (interquartile range) Pi Cramér‐Rao lower bounds was 30.1% (21.5 to 48.7)

### 3.4 Dilated cardiomyopathy patient

The patient with DCM underwent stress at 7T without complication or complaint and tolerated the protocol well, allowing for a recovery STEAM‐^31^P‐MRS scan (heart rate 64 → 108 → 64 beats/min) RPP 8970 → 13417 → 7680 [rest/stress/recovery]. Pi was robustly seen in all 3 scans. Data are presented in Figure 6 to demonstrate feasibility; resting PCr/ATP was unusually high for a DCM population recorded at 2.46, although within the range of values published in this population at this field strength.^4^ Pi/PCr increased fourfold during stress and fell back during recovery (stress/rest/recovery [0.076/0.268/0.119]; pH fell during stress (7.1/6.7/6.9).

## 4 DISCUSSION

As was our goal, STEAM‐^31^P‐MRS at 7T generated dark‐blood contrast with sufficient SNR to allow Pi peak demonstration, with minimal 2,3‐DPG contamination in human ^31^P spectra. The reasonable length of the scan meant that Pi estimation was also straightforward during stress. Although SNR, primarily due to low concentrations and the heart’s unfavourable geometry, limited our ability to measure Pi in 100% of scans, we report a technique whose repeatability competes with the slower CSI approach to Pi estimation^32^ and the computation of PCr/ATP both at 3T^48^ and 7T.^39^ The protocol is feasible for use in patients, which will be further investigated, and can be delivered during dobutamine infusion at 7T; indeed, our study is the first to deliver vasoactive stress during MR examinations at this field strength. We demonstrated no change in myocardial pH or Pi/PCr during dobutamine stress in healthy controls, which was well tolerated by all participants. Resting state PCr/ATP in our volunteers (2.1 ± 0.5) was comparable to recently published values at 7T of 2.1 ± 0.2^32^ and 1.9 ± 0.4,^39^ corroborating normal resting myocardial energetics in this cohort. We measured resting myocardial pH (7.07 ± 0.08) to be more acidic than blood pH (7.35‐7.45)^49^ consistent with that measured in other studies employing both a UTE‐CSI with long T_R_ method at 7T (7.12 ± 0.04^32^), or a ^1^H decoupling method at 1.5T (7.08 ± 0.03^33^) to quantity cardiac pH. Our reported P_i_/PCr of 0.095 ± 0.030 agrees with previous human cardiac studies of 0.097 ± 0.072^33^ and 0.11 ± 0.02.^32^

Myocardial Pi concentration is low; therefore, poor SNR reduces our ability to define Pi to a greater extent than other phosphorous containing metabolites during ^31^P‐MRS. In some cases in our study (4 of 41, pooled total rest scans) SNR prevented us from reliably quantifying Pi; these 4 cases had lower PCr SNR (values of 29, 37, 35, and 41) compared to the group average of 92. As is the case in human ^31^P‐MRS, anatomic variability is the primary reason; however, compared to gradient echo‐like sequences, STEAM sequences may suffer signal loss due to diffusion and myocardial motion. Indeed, during stress we did see a fall in SNR in some volunteers (which in 2 cases led to a failure to evaluate Pi with any certainty). This is likely due to extra motion (cardiac, respiratory, and also participant restlessness). SNR changes are, however, presumed to effect both interleaves equally, and when quantified Pi/PCr was unchanged and Pi Peak position (and thus measured pH) remains unaffected. Assessing myocardial Pi during stress is essential to uncover latent energetic deficits that may manifest in very large movements at higher workloads. As such, in rare cases, clear‐cut detection of Pi may be obvious only during stress. In these cases, a maximal Pi amplitude that would still not be determined above the noise floor can be generated using the fitting algorithm, and an estimate for Pi/PCr at rest generated (given adequate PCr SNR) to allow the stress response to be quantified but, importantly, not overestimated.

We report gross stability of myocardial Pi/PCr and pH during a twofold increase in work by the healthy heart. This result, although similar to the conservation of PCr/ATP under stress seen in healthy human subjects,^5^, ^7^, ^13^, ^14^ seems inconsistent with the hypothesis that movements in intracellular Pi are integral to ATP homeostasis as work increases. Indeed, an early ^31^P‐MRS experiment in healthy humans in which myocardial Pi was estimated semiquantitatively by assuming any change to the spectral resonance in the region of 2,3‐DPG during stress represents a change in Pi demonstrated an increase in Pi by ~33% during a threefold increase in the RPP.^50^ This study (in contrast to those above) also reported a concomitant fall in PCr/ATP. Our result is, however, consistent with preclinical work demonstrating stability of Pi (measured directly with NMR in open chest models at high field strength) as the heart rate increases with dobutamine.^30^, ^51^ In such models, higher levels of cardiac stress induced by high‐dose dobutamine, or uncoupling agents such as Dinitrophenol, do however induce detectable rises in Pi.^30^, ^52^ Computational models on available data,^19^, ^20^, ^53^ however, agree that Pi and ADP together feedback rates of cytosolic ATP hydrolysis to mitochondrial oxidative phosphorylation, and isolated mitochondria are indeed highly sensitive to small changes in Pi around the cardiac intracellular concentration.^54^ These changes involved in physiological signaling are perhaps too small to be detected by in vivo ^31^P‐MRS, which is the most appropriate interpretation of our result. The large rises seen in the animal models above likely demonstrate a loss of the steady state, not related to the control of respiration, at a point that we do not expose here with low‐grade stress in healthy volunteers. These animal models also demonstrate the point at which Pi begins to rise occurs at lower degrees of stress in conditions such as hypertrophy or heart failure.^30^, ^52^ Consistent with this is a model of human HCM, predicting a threefold increase in Pi during work in this condition^25^; we may be able to demonstrate this by studying patients with impaired energetics using our protocol. On rare occasions, we could not robustly see the Pi peak at rest; however, this would become less important if pathological energy metabolism resulted in similar clear‐cut increases of Pi concentration during stress. Our results do, however, suggest that gross movements in Pi, which would incur a change in cellular ΔG_ATP_, are not required for the control of oxidative phosphorylation.

As could be expected from the dark blood contrast produced during ^1^H MRI,^35^ any residual 2,3‐DPG signal was not distinguishable from noise by visual inspection in any of the spectra. 2,3‐DPG is an exclusively blood born red cell metabolite acting as an allosteric effector ensuring efficient tissue oxygen delivery,^55^ with none found within the myocyte itself. As such, the majority of 2,3‐DPG within the field of interest is within the ventricle, with a smaller amount found within the myocardial microcirculation. Both of these compartments are mobile, and although it is not possible to prove 100% suppression, we believe that ^31^P‐STEAM suppresses 2,3‐DPG sufficiently to allow robust in vivo quantification of Pi concentration. Further potential drawbacks of the technique should be considered. The approach was unique in suppressing signals from moving compartments, making it susceptible to some diffusion weighting. With a *b* value of 30 s/mm^2^ this, however, would lead to less than 5% proton signal attenuation given the myocardial diffusivity of 1.43 × 10^−3^ mm^2^/s,^56^ which would be lower for intracellular Pi and PCr. Spoiling gradients are an essential part of STEAM to remove residual unwanted magnetization (spurious FIDs and spin echoes) and were employed after each of the 3 pulses. These introduce motion sensitivity, and further adjustment of the spoilers could potentially improve SNR in future work. Our Pi data were not corrected for T_2_ relaxation because 7T cardiac ^31^P T_2_ values are not currently available. However, assuming similar T_2_ values to skeletal muscle,^57^ only a very small (6.6 %) underestimate in our ratios would result. Some considerations arise with the large single voxel used for STEAM MRS. Despite estimating transmit efficiency and adjusting coil voltages for each participant, inevitably some variation in flip angle (20%‐30%) will result over the voxel. This is, however, impossible to avoid with standard amplitude‐modulated selective pulses. The MRS sequences used in this study here are free breathing. The relatively large voxel size for STEAM makes uniform coverage of the septum more likely throughout the respiratory cycle compared to previously published 3D CSI protocols. Respiratory gating or retrospective “binning” and processing of spectra acquired at the end of expiration may reduce extraneous signals, and would, however, increase scan time to such an extent that studies during dobutamine stress would no longer be feasible.

The DCM case presented here is a demonstration of the feasibility of using this technique to study pathological energy metabolism and its acceptability to the patient studied. The result (Pi/PCr rise during stress, and a pH fall) is hypothesis generating. A properly powered follow‐on study will be needed to draw etiological conclusions. Animal evidence confirms that Pi rises with impaired CK flux,^22^ and Pi is predicted to rise during exercise in an energetic model of human HCM.^25^ We believe that our method, especially applied during stress (to expose latent deficits), will in the future offer a new window of understanding for conditions characterized by depressed CK reserve such as heart failure.^23^, ^24^ The ability to measure Pi/PCr in clinical studies of cardiac energetics both at rest and at stress has a number of further advantages over PCr/ATP. With loss of PCr and ATP seen as disease progresses,^8^, ^9^ along with homeostasis of [ATP] during the earlier stages of disease, Pi/PCr may be a more sensitive marker of energetic dysfunction than (PCr/ATP). Indeed, the increase in intracellular Pi in response to an increasing cardiac workload (pacing or dobutamine) is shown to be exaggerated in animal models of left ventricular hypertrophy compared to controls, a change detected earlier and with greater magnitude than a fall in PCr.^30^, ^52^ With Pi concentration, a direct contributor to the expression for ΔG_ATP_ and acting as a feedback signal to increase rates of ATP production by mitochondrial oxidative phosphorylation,^19^, ^20^ STEAM‐^31^P‐MRS may also be an appropriate and convenient candidate to further our understanding of these processes. Additional to energetics, Pi detection allows noninvasive myocardial pH assessment, which could prove useful (especially during the transition to stress) to detect the switch to anaerobic metabolism that defines ischaemia. Regional detection of such metabolic changes within the heart in distinct myocardial segments could be possible when combining the technique with a ^31^P‐MRS body coil, which gives circumferential ventricular signal.^58^ Use of a body^59^, ^60^ volume transmit coil may also provide increased RF bandwidth, mitigating the need for separate excitations and reducing potentially confounding effects of frequency fluctuations on the calculated pH.

## 5 CONCLUSION

7T STEAM‐^31^P‐MRS can measure cardiac Pi/PCr and pH at rest and during catecholamine stress. We clearly measure the inorganic phosphate peak and suppress 2‐3 DPG signal both at rest and during stress in a healthy cohort. The demonstration of feasibility in a patient with DCM motivates further investigation of the technique as a potential addition to future studies of pathological myocardial energetics.

## Supporting information


**FIGURE S1** Fitting of all peaks was performed using the open‐source Matlab‐based “OXSA” toolbox.^42^, ^43^ Here the direct output for fitting of the spectra from the Pi interleave is shown. Apodisation is applied for visualisation purposes. There is little left in the residuum (lower panel)
**FIGURE S2** Spectra demonstrating a significant loss of SNR during stress; no quantification of the effect of stress on Pi/PCr can be made, with only the rest data point used (participant 15)
**TABLE S1** Data from all volunteers in our study. Myocardial [Pi] is extremely low, thus poor SNR at rest or any significant loss of SNR as is commonly encountered during dobutamine stress, impacted our ability to see Pi. Data was included for analysis if the Pi peak was clearly resolved (defined as SNR > 2.5 and visually obvious). ✓ Denotes robust Pi peak (visually obvious and SNR> 2.5), × denotes no Pi resonance seen. An example of a case in which we saw a loss of SNR during stress can be seen in the Supporting Information Figure S1
