# Supplementary material for: Quantifying the effect of dobutamine stress on myocardial Pi and pH in healthy volunteers: A 31P MRS study at 7T
Source: Magn Reson Med. 2020 Sep 14;85(3):1147–59. doi: 10.1002/mrm.28494 (PMC8239988; doi:10.1002/mrm.28494)
Supplement: Supplementary file 1 — FIGURE S1 Fitting of all peaks was performed using the open‐source Matlab‐based “OXSA” toolbox. 42 , 43 Here the direct output for fitting of the spectra from the Pi interleave is shown. Apodisation is applied for visualisation purposes. There is little left in the residuum (lower panel) FIGURE S2 Spectra demonstrating a significant loss of SNR during stress; no quantification of the effect of stress on Pi/PCr can be made, with only the rest data point used (participant 15) TABLE S1 Data from all volunteers in our study. Myocardial [Pi] is extremely low, thus poor SNR at rest or any significant loss of SNR as is commonly encountered during dobutamine stress, impacted our ability to see Pi. Data was included for analysis if the Pi peak was clearly resolved (defined as SNR > 2.5 and visually obvious). ✓ Denotes robust Pi peak (visually obvious and SNR> 2.5), × denotes no Pi resonance seen. An example of a case in which we saw a loss of SNR during stress can be seen in the Supporting Information Figure S1 [file MRM-85-1147-s001.docx]

**Supplementary Data.**

**Supporting Information Table S1**

| Repeatability participant ID | Scan 1 Pi | Scan 2 Pi | Pi/PCr  (paired analysis) | pH  (paired analysis) |
| --- | --- | --- | --- | --- |
| 01 | ✓ | ✓ | ✓ | ✓ |
| 02 | ✓ | ✓ | ✓ | ✓ |
| 03 | ✓ | ✓ | ✓ | ✓ |
| 04 | ✓ | ✓ | ✓ | ✓ |
| 05 | ✓ | ✓ | ✓ | ✓ |
| 06 | ✓ | ✓ | ✓ | ✓ |
| 07 | ✓ | ✓ | ✓ | ✓ |
| 08 | × | ✓ | × | × |
| 09 | ✓ | ✓ | ✓ | ✓ |
|  |  |  |  |  |
|  |  |  |  |  |
|  |  |  |  |  |
| Stress Study participant ID | **Rest Pi** | **Stress Pi** | **Pi/PCr**  **(paired analysis)** | **pH**  **(paired analysis)** |
| 01 | ✓ | ✓ | ✓ | ✓ |
| 02 | ✓ | ✓ | ✓ | ✓ |
| 03 | × | × | × | × |
| 04 | ✓ | ✓ | ✓ | ✓ |
| 05 | ✓ | ✓ | ✓ | ✓ |
| 06 | ✓ | ✓ | ✓ | ✓ |
| 07 | ✓ | ✓ | ✓ | ✓ |
| 08 | ✓ | ✓ | ✓ | ✓ |
| 09 | ✓ | N/A | N/A | N/A |
| 10 | ✓ | ✓ | ✓ | ✓ |
| 11 | × | ✓ | × | × |
| 12 | ✓ | ✓ | ✓ | ✓ |
| 13 | ✓ | ✓ | ✓ | ✓ |
| 14 | ✓ | ✓ | ✓ | ✓ |
| 15 | ✓ | × | × | × |
| 16 | ✓ | × | × | × |
| 17 | × | × | × | × |
| 18 | ✓ | ✓ | ✓ | ✓ |
| 19 | ✓ | ✓ | ✓ | ✓ |
| 20 | ✓ | ✓ | ✓ | ✓ |
| 21 | ✓ | ✓ | ✓ | ✓ |
| 22 | ✓ | ✓ | ✓ | ✓ |
| 23 | ✓ | ✓ | ✓ | ✓ |

**Supporting Information Figure S1**

**
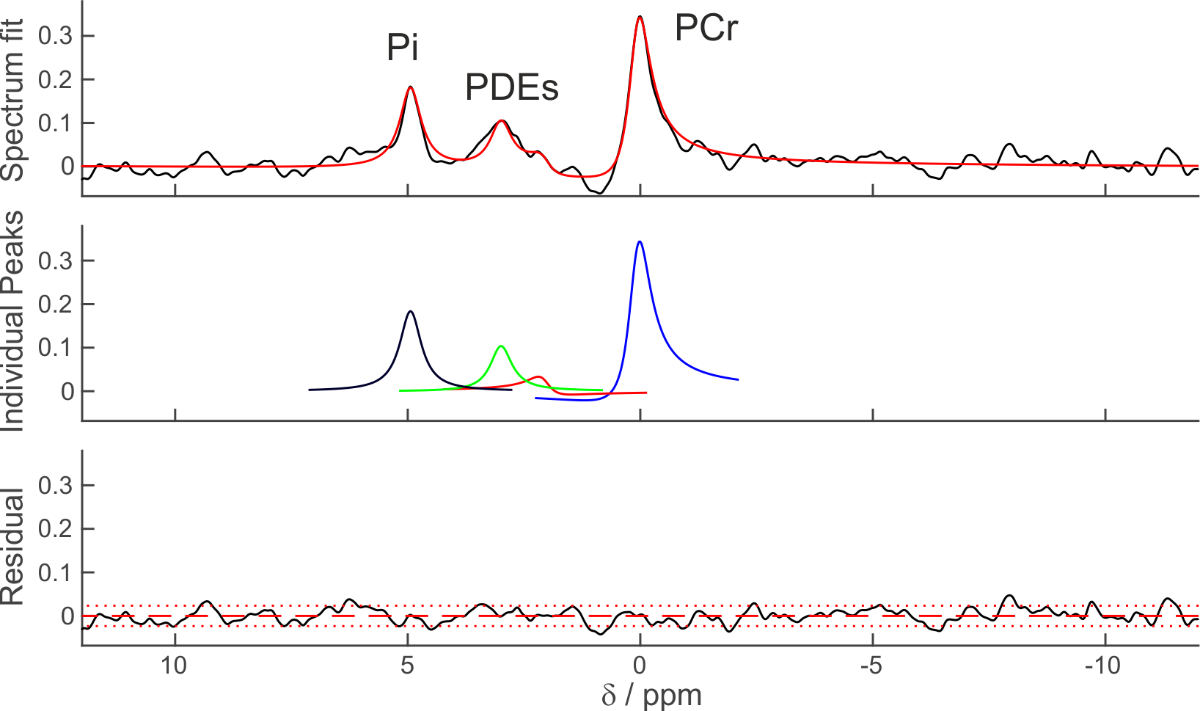
**

**Supporting Information Figure S2**

**
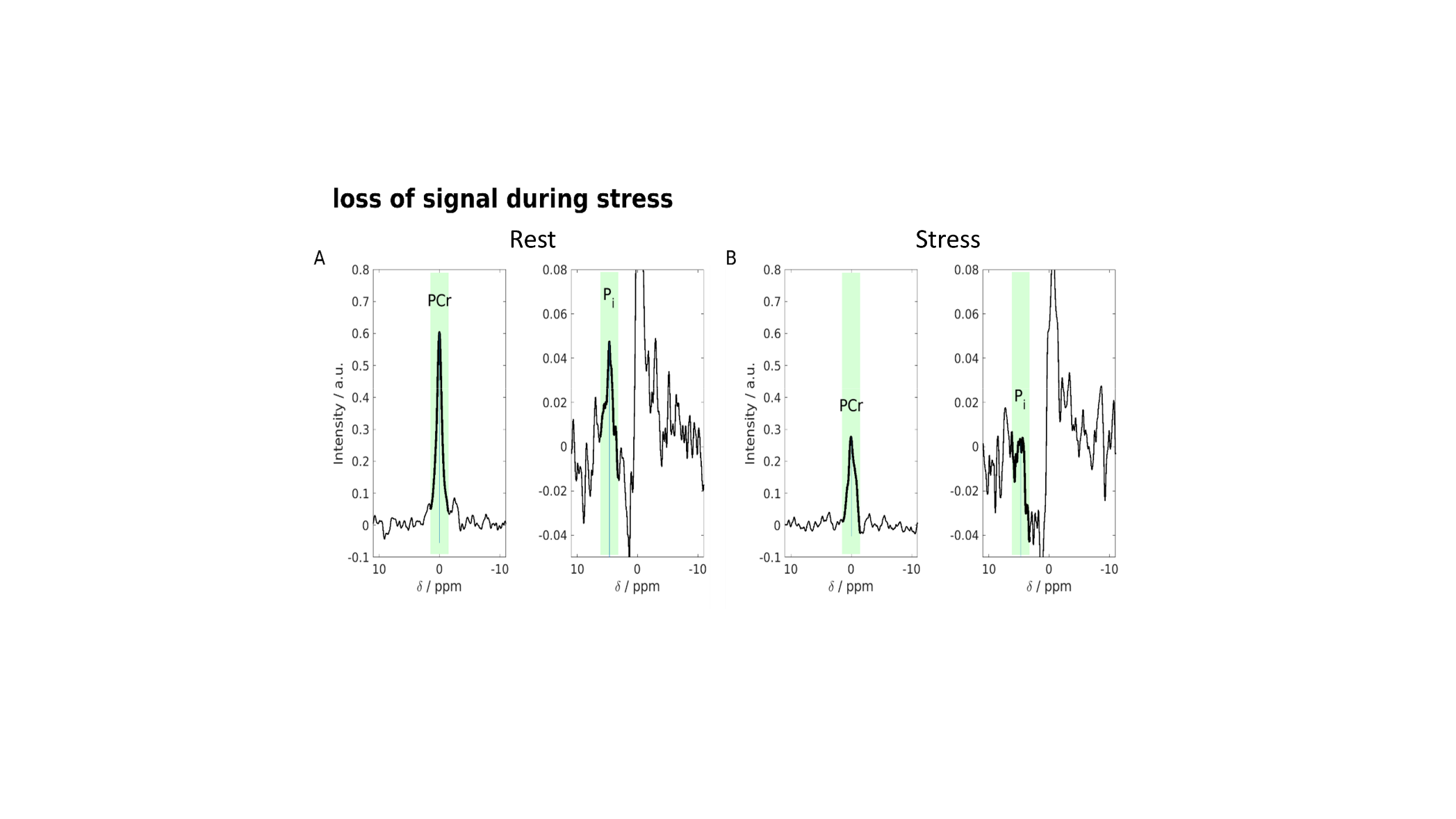
**
